# Severe Refractory Calcitriol-Mediated Hypercalcemia in Diffuse Large B-Cell Lymphoma Treated With Hemodialysis

**DOI:** 10.1210/jcemcr/luaf274

**Published:** 2025-12-03

**Authors:** Julius Wu, Gitanjali Reddy, Zhanna Zavgorodneva, Lisel Hope

**Affiliations:** College of Medicine, SUNY Downstate Health Sciences University, Brooklyn, NY 11203, USA; College of Medicine, SUNY Downstate Health Sciences University, Brooklyn, NY 11203, USA; Department of Endocrinology, Diabetes, and Metabolism, SUNY Downstate Health Sciences University, Brooklyn, NY 11203, USA; Department of Endocrinology, Diabetes, and Metabolism, SUNY Downstate Health Sciences University, Brooklyn, NY 11203, USA

**Keywords:** hypercalcemia of malignancy, non-Hodgkin lymphoma, diffuse large B-cell lymphoma, calcitriol

## Abstract

Hypercalcemia of malignancy is a recognized complication of diffuse large B-cell lymphoma (DLBCL) and is associated with decreased survival time and more advanced disease. We present a case of DLBCL complicated by severe refractory calcitriol-mediated hypercalcemia and renal failure that was unresponsive to conventional treatments. Treatment with glucocorticoids and concurrent antiresorptive therapy was also ineffective. Ultimately, hemodialysis was necessary to treat the patient's hypercalcemia and tenuous volume status. This case demonstrates that calcitriol-mediated hypercalcemia of malignancy in DLBCL may be unresponsive to standard hypercalcemia interventions, and it supports the utility of hemodialysis as a method of treating hypercalcemia, especially in patients with renal impairment.

## Introduction

Hypercalcemia is a common complication of malignancy, affecting as many as 20% to 30% of cancer patients at some point during their disease course [1]. Hypercalcemia is often a consequence of late-stage cancers and is associated with a poor prognosis [2]. Several mechanisms underlie hypercalcemia of malignancy. Humoral hypercalcemia, the most common overall form, results from tumor parathyroid hormone-related protein (PTHrP) secretion causing bone resorption and increased calcium reabsorption at the distal tubules [1]. It is most commonly seen in squamous cell carcinomas of the lung, head, and neck, urothelial carcinomas, breast carcinomas, and rarely non-Hodgkin lymphoma [3]. Hypercalcemia can also arise in the setting of osteolytic metastases, where tumor-produced cytokines induce osteoclastic bone resorption and inhibit osteoblastic bone formation [4]. This is most frequently seen in solid tumors that metastasize to bone, such as breast cancer and multiple myeloma [2].

A less common cause of hypercalcemia of malignancy is extrarenal production of 1,25-dihydroxyvitamin D leading to increased intestinal absorption of calcium and, to a lesser degree, increased bone resorption. This mechanism is more frequently seen in human T-lymphotropic virus 1 (HTLV-1)-associated adult T-cell leukemia/lymphoma, as well as Hodgkin and non-Hodgkin lymphomas [5]. Nevertheless, 1,25-dihydroxyvitamin D-mediated hypercalcemia of malignancy remains rare overall, accounting for less than 1% of cases [2]. Here, we present a case of diffuse large B-cell lymphoma (DLBCL), germinal-center B-cell subtype, complicated by severe refractory calcitriol-mediated hypercalcemia resistant to conventional hypercalcemia treatments.

## Case Presentation

An 80-year-old woman with a past medical history of diabetes mellitus, stage 3 chronic kidney disease, hypertension, and hyperlipidemia presented to the emergency department with dizziness and unsteady ambulation of 4 days’ duration. She additionally reported cramping lower abdominal pain for 3 weeks associated with constipation, and bilateral hip pain radiating to the knees for 1 month. She was accompanied by her son, who endorsed a change in mental status over 2 weeks, noting that she had confusion, memory difficulties, and slurred speech. She denied chest pain, shortness of breath, cough, fever, nausea/vomiting, headache, or visual disturbances.

The patient's home medications included amlodipine 10 mg daily, atorvastatin 40 mg daily, empagliflozin 10 mg daily, gabapentin 100 mg daily, hydrochlorothiazide 25 mg daily, metoprolol succinate 50 mg daily, and valsartan 320 mg daily. She did not take any calcium or vitamin D supplements, and she had a balanced diet with a limited intake of dairy products.

On physical examination, the patient was alert and oriented to person and place but not time. Her oral mucosa appeared dry, and she had a distended abdomen with a palpable mass. Cardiac and pulmonary exams were unremarkable, and the neurological exam did not reveal any focal deficits.

## Diagnostic Assessment

Initial laboratory workup was notable for a markedly elevated total calcium of 17.7 mg/dL (SI: 4.425 mmol/L) (reference range: 8.8-10.2 mg/dL [SI: 2.1-2.6 mmol/L]), ionized calcium of 9.04 mg/dL (SI: 2.26 mmol/L), (reference range: 4.5-5.6 mg/dL [SI: 1.16-1.32 mmol/L]), and albumin of 3.6 g/dL (SI: 36 g/L) (reference range: 3.5-5.5 g/dL [SI: 35-55 g/L]). Phosphate and magnesium were normal. The parathyroid hormone (PTH) was inappropriately normal at 51.2 pg/mL (SI: 5.43 pmol/L), (reference range: 15.0-65.0 pg/mL [SI: 1.6-6.9 pmol/L]) while 25-hydroxyvitamin D was decreased at 26 ng/mL (SI: 65 nmol/L), (reference range: 30-50 ng/mL [SI: 60-125 nmol/L]) and 1,25-hydroxyvitamin D was elevated at 112 pg/mL (SI: 279.55 pmol/L), (reference range: 19.9-79.3 pg/mL [SI: 59-159 pmol/L]). PTHrP was less than 2.0 pmol/L. Thyroid-stimulating hormone (TSH) and free T4, serum and urine electrophoresis, and angiotensin-converting enzyme (ACE) were all normal. Other biomarkers on admission are summarized in Table 1.

**Table 1.** Patient laboratory investigations upon admission

Investigation	Result (reference range)	
	Conventional units	Système international (SI) units
*Electrolytes and protein*		
Calcium	17.7 mg/dL (8.6-10.3 mg/dL)	4.425 mmol/L (2.1-2.6 mmol/L)
Calcium ionized	9.04 mg/dL (4.5-5.6 mg/dL)	2.26 mmol/L (1.12-1.32 mmol/L)
Albumin	3.6 g/dL (3.4-5.4 g/dL)	36 g/L (35-55 g/L)
Total protein	6.8 g/dL (6.0-8.3 g/dL)	68 g/L (60-83 g/L)
Phosphate	3.5 mg/dL (2.5-4.5 mg/dL)	1.13 mmol/L (0.81-1.45 mmol/L)
Magnesium	2.2 mg/dL (1.7-2.3 mg/dL)	0.91 mmol/L (0.7-0.95 mmol/L)
Sodium	142 mmol/L (135-145 mmol/L)	
Potassium	4.1 mmol/L (3.5-5.1 mmol/L)	
Chloride	104 mmol/L (98-107 mmol/L)	
*Endocrine*		
TSH	2.58 µIU/mL (0.450-4.500 µIU/mL)	2.58 mIU/L (0.450-4.500 mIU/L)
Free T4	1.5 ng/dL (0.82-1.77 ng/dL)	19.3 pmol/L (10.6-22.8 pmol/L)
PTH	51.2 pg/mL (15.0-65.0 pg/mL)	5.43 pmol/L (1.6-6.9 pmol/L)
PTHrP	<2.0 pmol/L (<2.0 pmol/L)	
25-hydroxyvitamin D	26 ng/mL (30-50 ng/mL)	65 nmol/L (60-125 nmol/L)
1,25-hydroxyvitamin D	112 pg/mL (19.9-79.3 pg/mL)	279.55 pmol/L (59-159 pmol/L)
*Renal and hepatic*		
BUN	79 mg/dL (7-20 mg/dL)	28.2 mmol/L (2.50-7.14 mmol/L)
Creatinine	3.5 mg/dL (0.70-1.30 mg/dL)	309.47 μmol/L (61.9-114.9 μmol/L)
eGFR	12.7 mL/min/1.73 m^2^ (>60 mL/min/1.73 m^2^)	
ALT	46 U/L (0-44 U/L)	
AST	75 U/L (0-40 U/L)	
Alkaline phosphatase	223 U/L (44-147 U/L)	
*Hematologic*		
WBC	6.72*10^3^/µL (4.0-10.5*10^3^/µL)	6.72*10^9^/L (4.0-10.5*10^9^/L)
Hemoglobin	10 g/dL (12.0-16.0 g/dL)	6.21 mmol/L (7.45-9.93 mmol/L)
Hematocrit	33.1% (36.0-46.0%)	0.33 (0.36-0.46)
MCV	83.4 fL (80-100 fL)	
Platelets	127*10^3^/µL (150-400*10^3^/µL)	127*10^9^/L (150-400*10^9^/L)
*Other studies*		
Venous lactate	3.5 mmol/L (0.5-2.2 mmol/L)	
High-sensitivity troponin T	60 ng/L (<14 ng/L)	

Abbreviations: ALT, alanine aminotransferase; AST, aspartate aminotransferase; BUN, blood urea nitrogen; eGFR, estimated glomerular filtration rate; MCV, mean corpuscular volume; PTH, parathyroid hormone; PTHrP, parathyroid hormone-related peptide; T4, thyroxine; TSH, thyroid-stimulating hormone; WBC, white blood cells.

The creatinine level was 3.5 mg/dL (SI: 309.47 μmol/L), (reference range: 0.5-0.9 mg/dL [SI: 61.9-114.9 mmol/L]), an increase from the patient's baseline of 1.17 mg/dL (SI: 103.45 μmol/L) measured 2 months prior, indicating acute kidney injury (AKI). Estimated glomerular filtration rate (eGFR) had also decreased to 12.7 mL/min/1.73 m^2^ (reference range: >60 mL/min/1.73 m^2^) from 47.0 mL/min/1.73 m^2^ 2 months earlier. Venous lactate was 3.5 mmol/L (reference range: 0.6-1.4 mmol/L) and high-sensitivity troponin T was 60 ng/L (SI: 0.06 ng/mL), (reference range: 0-14 ng/L [SI: 0-0.014 ng/mL]). An electrocardiogram revealed T-wave inversions without ischemic changes. Computed tomography (CT) of the head was negative for acute findings.

A CT of the abdomen and pelvis demonstrated a massively enlarged uterus, multiple large hypoattenuating hepatic lesions, and right hemithorax subpleural nodules concerning for metastases.

Due to the imaging findings, hypercalcemia of malignancy was suspected, and the patient underwent core needle biopsy of one of the liver lesions. The biopsy revealed DLBCL, germinal-center B-cell subtype, with a Ki-67 proliferation index of 50% to 60%. Immunohistochemical stains of the neoplastic cells were positive for CD20, BCL6, and CD30 and negative for CD3, CD10, CD56, BCL1, BCL2, MUM1, and c-MYC. In situ hybridization for Epstein-Barr virus encoded RNA was negative.

The patient's inappropriately normal PTH also raised suspicion for concurrent primary hyperparathyroidism. She underwent a parathyroid ultrasound, which revealed a 1.98-cm well-defined round solid nodule suggestive of a parathyroid adenoma. However, further workup with a Sestamibi scan did not demonstrate persistent uptake, and a follow-up 4D CT of the parathyroids confirmed the absence of a parathyroid adenoma.

## Treatment

The patient was admitted to the hospital for hypercalcemia treatment and malignancy workup. Her home hydrochlorothiazide was stopped. While the malignancy workup was pending, she underwent multiple interventions to treat her hypercalcemia. She began receiving intravenous fluids, cinacalcet 30 mg twice daily, and calcitonin at 4 units/kg. Over the subsequent weeks, the patient's AKI showed no improvement, with serum creatinine levels persisting between 2 and 3 mg/dL (SI: 176.84-265.26 μmol/L). Bisphosphonates were contraindicated due to the patient's renal function. Due to her kidney dysfunction and risk of volume overload, furosemide was added to the regimen.

Following these interventions, the corrected calcium decreased but remained elevated at 13.8 mg/dL (SI: 3.44 mmol/L) after completing the first round of calcitonin. She then received denosumab 120 mg subcutaneously, and the cinacalcet was increased to 60 mg twice daily. She remained hypercalcemic and continued to be treated with intermittent calcitonin at 8 units/kg and denosumab. She also received hydrocortisone 200 mg for 4 days. However, the calcium continued to rise, reaching a maximum of 18.5 mg/dL (SI: 4.62 mmol/L) (corrected) 2 days after she completed the course of hydrocortisone. Also, the intravenous fluids led to the complication of fluid overload, which resulted in pulmonary edema and dyspnea.

## Outcome and Follow-Up

**Figure 1.**
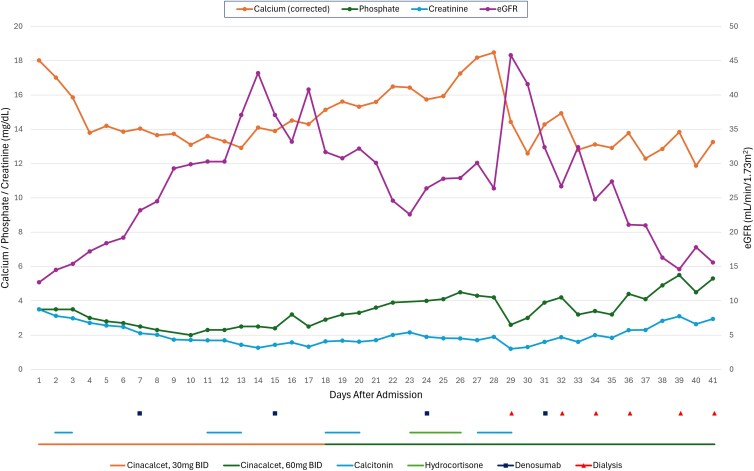
Calcium, phosphate, creatinine, and eGFR trends following admission and hypercalcemia treatments.

Over the entire hospital course, the patient received 4 rounds of calcitonin, 4 doses of denosumab 120 mg, 4 days of hydrocortisone 200 mg, and daily cinacalcet. The patient's calcium, phosphate, creatinine, and eGFR trends, and timing of hypercalcemia treatments, are summarized in Fig. 1. Due to the severe refractory hypercalcemia and fluid status, the patient was started on dialysis using low-calcium dialysate. Following her first session, the corrected calcium decreased to 12.6 mg/dL (SI: 3.14 mmol/L). Subsequent post-dialysis corrected calcium levels were in the range of 11.9-12.9 mg/dL (SI: 2.97-3.22 mmol/L). After receiving the liver biopsy pathology results, systemic therapy with rituximab and reduced-dose cyclophosphamide, doxorubicin, vincristine, and prednisone (R-mini-CHOP) was initiated. With these interventions, the calcium normalized, and the patient was discharged. At 2-month follow-up, serum calcium remained normal, the patient continued systemic chemotherapy and remained on thrice-weekly hemodialysis. Hemodialysis served as a bridge to allow safe administration of chemotherapy. Whether dialysis can be discontinued after completion of chemotherapy remains to be determined.

## Discussion

Our case demonstrates that hypercalcemia in DLBCL may be severe and refractory to conventional treatments. Hypercalcemia is uncommonly seen in non-Hodgkin lymphoma (NHL), with previous studies reporting an incidence between 7.1% and 13% [6-8]. However, hypercalcemia appears to be more common among DLBCL specifically, with the prevalence at diagnosis reported between 18% and 23% [9, 10]. In NHL, hypercalcemia is associated with higher-grade cancers, with incidences ranging from 1% to 2% in low-grade NHL to up to 30% in high-grade disease [5, 7]. In general, hypercalcemia of malignancy is associated with a poor prognosis, even among those receiving treatment for their cancer [11]. In one study examining the prognostic significance of hypercalcemia in B-cell NHL, the median survival of patients presenting with hypercalcemia was only 9 months [7]. In DLBCL, hypercalcemia is also associated with shorter overall survival times and more advanced disease at presentation [9, 10].

Elevated calcitriol production is one of the major mechanisms of hypercalcemia in NHL [12], though there are case reports of PTHrP-mediated hypercalcemia as well [13-15]. However, the cause is not always identified: a more recent study in a group of hypercalcemic NHL patients found that most had neither elevated 1,25-hydroxyvitamin D nor PTHrP levels, suggesting another mechanism by which hypercalcemia occurs in this population [16]. In our case, the patient's hypercalcemia was mediated by elevated 1,25-hydroxyvitamin D. Workup for other causes of hypercalcemia, such as PTHrP secretion, bony metastases, and parathyroid adenoma, was negative. Thiazide diuretics, which the patient had been taking prior to hospitalization, may have also contributed to the development of severe hypercalcemia. However, with rare exceptions, thiazide-associated hypercalcemia is usually mild and transient.

A number of different treatments for hypercalcemia of malignancy exist, aimed at correcting hypovolemia, decreasing bone resorption, increasing renal calcium excretion, and addressing the underlying malignancy. Initial management includes intravenous normal saline, which treats hypercalcemia-induced dehydration and encourages renal calcium excretion via increasing sodium delivery to the distal tubules [2]. Loop diuretics are also occasionally used, as in this case where the patient's poor renal function predisposed her to volume overload, but their use in the primary treatment of hypercalcemia has been called into question [17]. Antiresorptive therapy has emerged as the crux of treatment for hypercalcemia of malignancy and encompasses the bisphosphonates, calcitonin, and denosumab. Of these, bisphosphonates have formed part of the standard treatment for hypercalcemia; however, they have been associated with adverse renal effects and their use is not recommended in patients with a creatinine clearance of <30 to 35 mL/min [18, 19]. In this case, the patient's renal status precluded the use of bisphosphonates, leading us to pursue other treatments instead.

Glucocorticoids are a notable avenue of treatment for patients with calcitriol-mediated hypercalcemia. Their efficacy lies in their inhibition of 1α-hydroxylase, which decreases the conversion of 25-hydroxyvitamin D to 1,25-hydroxyvitamin D [20]. However, evidence supporting glucocorticoids’ efficacy in treating hypercalcemia in NHL, suggested dosage and timing, and use in conjunction with other hypercalcemia treatments is currently limited. Current Endocrine Society guidelines support the use of glucocorticoids to treat calcitriol-mediated hypercalcemia of malignancy [21], though with the caveat that reductions in calcium levels are often transient. They also recommend addition of an intravenous bisphosphonate or denosumab in patients receiving glucocorticoids with continued severe hypercalcemia, although this recommendation was based on indirect evidence [21]. Our case is notable in that treatment with glucocorticoids did not result in any decrease, even temporary, in the patient's serum calcium. Furthermore, concurrent treatment with denosumab did not have a beneficial effect either. This indicates that glucocorticoids are not always effective in treating calcitriol-mediated hypercalcemia of malignancy, even in combination with antiresorptive therapy, especially when the degree of hypercalcemia is severe.

Managing hypercalcemia in the context of declining kidney function and tenuous volume status can be particularly challenging. Hydration can help correct prerenal AKI and decrease passive calcium reabsorption in the proximal tubule, while denosumab acts to inhibit skeletal calcium mobilization. However, when renal function is severely compromised, the mobilized calcium cannot be adequately excreted, even with the use of furosemide, resulting in persistent hypercalcemia. In the end, our patient's hypercalcemia was responsive to hemodialysis. Hemodialysis using low- or no-calcium dialysate has previously been shown to be effective in treating hypercalcemia due to multiple causes [22-24], including hypercalcemia of malignancy [25, 26]. Our case supports the utility of dialysis as a therapeutic option for refractory hypercalcemia of malignancy, particularly in the setting of renal failure where other treatments, such as bisphosphonates, are contraindicated. In these cases, dialysis may provide an important temporizing measure while workup and treatment for the underlying malignancy are pending. Finally, our case demonstrates the need for an individualized approach to hypercalcemia of malignancy. Although there was an identifiable mechanism of hypercalcemia in this case due to elevated calcitriol, targeted treatment with glucocorticoids was ineffective. This case highlights that hypercalcemia in DLBCL may behave unpredictably and be unresponsive to conventional treatments for hypercalcemia.

## Learning Points

- DLBCL is associated with calcitriol-mediated hypercalcemia of malignancy, which is linked to a poorer prognosis.
- Antiresorptive therapies such as bisphosphonates are first-line treatments for hypercalcemia but may be contraindicated in patients with significant renal dysfunction.
- Hemodialysis with low-calcium dialysate is a potential option to treat severe hypercalcemia that is refractory to conventional treatments (eg, fluids, antiresorptive therapy). Severe hypercalcemia may present with a variety of signs and symptoms such as altered mental status, abdominal pain, and renal impairment.
- Initiation of hemodialysis should be considered once it is clear that conventional therapy is insufficient to address hypercalcemia (typically evident at 1-2 weeks after initiation), especially if there are signs of worsening kidney function or volume overload.

## Contributors

All authors made individual contributions to authorship. J.W., G.R., Z.Z., and L.H. were involved in the diagnosis, management, and follow-up of the patient. J.W., G.R., and Z.Z. were involved in writing the manuscript. L.H. supervised the preparation of the manuscript and provided critical revisions to the final version. All authors reviewed and approved the final draft.

## Data Availability

Original data generated and analyzed during this study are included in this published article.

## Abbreviations

AKI: acute kidney injury
CT: computed tomography
DLBCL: diffuse large B-cell lymphoma
eGFR: estimated glomerular filtration rate
NHL: non-Hodgkin lymphoma
PTH: parathyroid hormone
PTHrP: parathyroid hormone-related protein
